# Dihydrobenz[*e*][1,4]oxazepin-2(3*H*)-ones, a new anthelmintic chemotype immobilising whipworm and reducing infectivity *in vivo*

**DOI:** 10.1371/journal.pntd.0005359

**Published:** 2017-02-09

**Authors:** Frederick A. Partridge, Emma A. Murphy, Nicky J. Willis, Carole J. R. Bataille, Ruth Forman, Narinder Heyer-Chauhan, Bruno Marinič, Daniel J. C. Sowood, Graham M. Wynne, Kathryn J. Else, Angela J. Russell, David B. Sattelle

**Affiliations:** 1 Centre for Respiratory Biology, UCL Respiratory, Division of Medicine, University College London, London, United Kingdom; 2 Faculty of Biology, Medicine and Health, University of Manchester, Manchester, United Kingdom; 3 Department of Chemistry, Chemistry Research Laboratory, University of Oxford, Oxford, United Kingdom; 4 Department of Pharmacology, University of Oxford, Oxford, United Kingdom; George Washington University, UNITED STATES

## Abstract

*Trichuris trichiura* is a human parasitic whipworm infecting around 500 million people globally, damaging the physical growth and educational performance of those infected. Current drug treatment options are limited and lack efficacy against the worm, preventing an eradication programme. It is therefore important to develop new treatments for trichuriasis. Using *Trichuris muris*, an established model for *T*. *trichiura*, we screened a library of 480 novel drug-like small molecules for compounds causing paralysis of the *ex vivo* adult parasite. We identified a class of dihydrobenz[*e*][1,4]oxazepin-2(3*H)*-one compounds with anthelmintic activity against *T*. *muris*. Further screening of structurally related compounds and resynthesis of the most potent molecules led to the identification of 20 active dihydrobenzoxazepinones, a class of molecule not previously implicated in nematode control. The most active immobilise adult *T*. *muris* with EC_50_ values around 25–50μM, comparable to the existing anthelmintic levamisole. The best compounds from this chemotype show low cytotoxicity against murine gut epithelial cells, demonstrating selectivity for the parasite. Developing a novel oral pharmaceutical treatment for a neglected disease and deploying it via mass drug administration is challenging. Interestingly, the dihydrobenzoxazepinone OX02983 reduces the ability of embryonated *T*. *muris* eggs to establish infection in the mouse host *in vivo*. Complementing the potential development of dihydrobenzoxazepinones as an oral anthelmintic, this supports an alternative strategy of developing a therapeutic that acts in the environment, perhaps via a spray, to interrupt the parasite lifecycle. Together these results show that the dihydrobenzoxazepinones are a new class of anthelmintic, active against both egg and adult stages of *Trichuris* parasites. They demonstrate encouraging selectivity for the parasite, and importantly show considerable scope for further optimisation to improve potency and pharmacokinetic properties with the aim of developing a clinical agent.

## Author summary

*Trichuris trichiura* is a human parasitic whipworm infecting around 500 million people globally and having major consequences on the physical growth and educational performance of those infected. Current drug treatment options are limited and lack efficacy against the worm. Critically, they lack the effectiveness that would allow for a practical program for eradication of this parasite. It is therefore important to develop new treatments for trichuriasis. We screened for molecules that could paralyse the adult of a closely related mouse parasite, and identified a class of compounds, the dihydrobenzoxazepinones, not previously implicated as anthelmintics. Importantly, our compounds are active against the parasite but show only low toxicity against mouse cells, demonstrating selectivity for the parasite. Dihydrobenzoxazepinones could be developed as potential pharmaceutical treatments for trichuriasis. Since developing and deploying new drugs for neglected diseases by mass administration is challenging, we also explored whether the compounds could potentially be used to interrupt the *Trichuris* lifecycle by acting on eggs. Our dihydrobenzoxazepinone compounds reduced the ability of *T*. *muris* eggs to establish infection in their mouse host. This supports an environmental spray strategy for the control of *Trichuris* targeting their eggs in environmental hotspots such as latrines.

## Introduction

Gastrointestinal nematode infections result in morbidity in humans and livestock; approximately 1 billion people worldwide are infected [1]. Trichuriasis, a neglected human tropical disease caused by infection by the whipworm *Trichuris trichiura*, affects approximately 500 million people mainly in developing countries, resulting in disability and poor child development [2]. Current strategies for helminth control include Mass Drug Administration (MDA) programs during childhood [2–5]. MDA typically uses benzimidazole anthelmintics against *Trichuris*, such as albendazole and mebendazole, but both show poor efficacy, with low cure rates and increasing drug resistance; similar problems are encountered when these compounds are used to target livestock *Trichuris* species [2,6]. Benzimidazoles inhibit microtubule synthesis, normally resulting in parasite immobilization [7] but unlike luminal dwelling nematodes such as *Ascaris*, where annual treatment with albendazole or mebendazole has resulted in cure rates of up to 95% [8], *Trichuris* is less well controlled, possibly due to the burrowing of the whipworm into intestinal epithelial cells, which may provide some protection from drugs. Modelling studies have concluded that MDA with benzimidazoles is unable to interrupt transmission of *T*. *trichiura* and therefore fails to achieve elimination in many settings [9]. Co-administration of two or more anthelmintics, such as albendazole plus ivermectin [10] or albendazole plus oxantel pamoate [10,11] has been demonstrated in randomised controlled trials to improve single-dose cure rates, and a recent study found that oxantel pamoate alone gave a single-dose cure rate of 60% at the optimal dose [12]. However even these improved cure rates are much lower than those achieved for other intestinal nematodes and again modelling suggests that they are insufficient to achieve elimination in higher transmission settings [9]. There are also concerns about the emergence of resistance [8,13]. No vaccine currently exists for trichuriasis. Elimination of this disease will likely require a three-way approach of new, more effective drugs, vaccine development, and complementary interventions in hygiene, sanitation and education.

Given the need for new classes of anti-*Trichuris* compounds, there have been a number of assays developed to screen compounds for activity against this parasite. These include manual scoring of motility; indirect assessment of viability by using the xCELLigence System; assessment of metabolic activity via colorimetric assays such as resazurin, MTT, and acid phosphatase activity; and assessment of motor activity via isothermal microcalorimetry [14–17].

A variety of plant materials have been shown to have activity against *Trichuris*. Koné and colleagues tested a large panel of plant extracts that are used in the Côte d’Ivoire to treat parasitic diseases for their activity against *T*. *muris* [18]. Notably, an extract from the leaves of *Combretum mucronatum* both blocked the motility of *T*. *muris* adults *in vitro* and was effective at reducing worm burden when used to treat mice infected with *T*. *muris*. Papaya latex (which is rich in cysteine proteases) or the purified proteases ficin and papain rapidly reduce motility of *T*. *muris* parasites *in vitro*, and this is associated with damage to nematode cuticle [19]. Indeed papaya latex extract is also effective at reducing the worm burden of mice infected with *T*. *muris*. These cysteine proteases from papaya have also been demonstrated in randomized control trials to be more effective than albendazole at treating *T*. *suis* in pigs [20]. However the large doses required for efficacy, of the order of 10-40g of protease, will be challenging for treating human patients. Recently, luteolin (a compound isolated from the Bhutanese daisy, *Ajania nubigena*), bergapten and isomyristicin (both isolated from *Pleurospermum amabile)* have all been shown to have activity against *T*. *muris*, in each case associated with cuticular damage [21,22].

**Fig 1:**
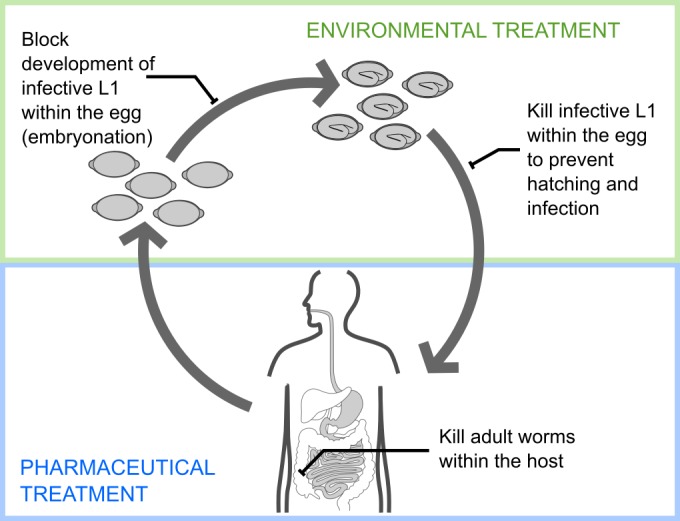
Schematic showing possible treatment strategies: post-infection treatment and breaking the *Trichuris* life cycle in the environment. *Trichuris* infection could be prevented by using novel drugs to 1) target the prevention of egg embryonation in the external environment, 2) reduce the infectivity of embryonated eggs prior to ingestion, or 3) treat existing infections *in vivo* while worms are at larval and adult stages.

The lifecycle of *T*. *trichiura* commences with unembryonated eggs being shed in the faeces of infected individuals. These eggs embryonate in the soil, thus becoming infective, and are transmitted to their human host by consumption of contaminated food, water or soil. The eggs hatch in the large intestine, and infective larvae invade the mucosal epithelium. After a series of larval moults, adult worms develop and release eggs into the lumen of the caecum. Several life cycle stages of *Trichuris* have the potential as viable targets for small molecule drugs including immobilisation or killing of worms inside the host following infection; preventing egg embryonation in the external environment; and reducing the infectivity of embryonated eggs prior to ingestion (Fig 1). Thus in addition to post-infection eradication of existing infections, the targeting of eggs and embryonation may break the parasite life-cycle and thereby offer potential for infection control strategies that avoid costly and impractical pharmaceutical development (e.g. an environmental spray targeting eggs in environmental hotspots such as latrines).

*T*. *muris*, is widely used as a laboratory model for investigations on trichuriasis, and severe combined immune deficiency (SCID) mice have been used as a system for culture of *T*. *muris* and for *in vivo* testing of the capacity of anthelmintic drug candidates to clear infection in the absence of adaptive immunity [23]. Given the close similarity between *T*. *muris* and *T*. *trichiura* at both the genetic and proteomic level [24] we embarked on a programme of drug discovery searching for new small molecules active against *T*. *muris* with a view to generating anthelmintic leads. Here we present our discovery of the dihydrobenzoxazepinones, a new class of molecules active against *T*. *muris*. These molecules paralyse the adult parasite, and also reduce the ability of treated eggs to establish infection in the host *in vivo*.

## Methods

### Ethics statement

All animal experiments were approved by the University of Manchester Animal Welfare and Ethical Review Board and performed under the regulation of the United Kingdom Home Office Scientific Procedures Act (1986) and the Home Office project licence 70/8127.

### *In vivo* culture of *Trichuris muris*

Severe combined immune deficiency (SCID) mice were bred in the Biological Services Facility at the University of Manchester. Both male and female SCID mice were infected with a high dose of 200 infective embryonated *T*. *muris* eggs via oral gavage. At day 35 post-infection, groups of 5 mice were killed and their caecae and colons removed. Guts were opened longitudinally, washed with pre-warmed Roswell Park Memorial Institute (RPMI) 1640 media supplemented with penicillin (500U/ml) and streptomycin (500μg/ml) and adult *T*. *muris* worms gently removed using fine forceps.

### *Ex vivo T*. *muris* adult maintenance for motility screen

Worms removed from SCID mice were maintained in RPMI-1640 media supplemented with penicillin (500U/ml) and streptomycin (500μg/ml) at approximately 37°C and studied on the same day. Using fine forceps, individual live worms were placed into 96 well plates containing 75μl of RPMI-1640 medium supplemented with penicillin (500U/ml) and streptomycin (500μg/ml) plus 1% final concentration of DMSO or compound dissolved in DMSO. In the primary screen the test compound concentration was 100μM. Plates were then incubated at 37°C, 5% CO_2_, and motility was analysed after 24 hours.

### Automated motility assay

Worm movement was quantified using an automated motility assay. This system gives an estimate of worm movement in each well by thresholding and quantifying pixel intensity variance over time. An earlier version of this system has been previously described [25,26]. One adult *T*. *muris* worm was used per well, with one well per compound in each replicate of the primary screen. The primary screen was repeated each week for a period of four weeks giving four independent biological replicates from separate parasite batches for each compound. Dose-response curves were calculated with the four factor log-logistic model using the R package *drc* [27].

### Small molecule compound library

The compound library used in this study (Chemistry Research Laboratory, University of Oxford) consists of over 50,000 drug-like small molecules, which has been assembled from a range of external and internal (proprietary) sources. A pilot screen was undertaken with a 480 compound subset of the main library, containing a variety of chemical scaffolds which were considered to be particularly amenable to further medicinal chemistry optimisation. This pilot screening set comprised more than twenty different mono- and polycyclic heteroaromatic scaffolds, each of which was substituted with a range of functional groups. Compounds were provided as 10mM solutions in DMSO and frozen at -20°C until required.

The primary screen was carried out at 100μM final concentration. Assay plates were assigned unique IDs and statistical analysis was performed without knowledge of compound identities using R scripts. Once hit wells were identified, well/plate positions were mapped to compound identities using DataWarrior [28]. Compounds active in the primary screen were retested in a blind secondary screen (n = 5, 100μM). Compounds identified as having encouraging activity from the DMSO solution samples were then resynthesized for confirmatory testing.

### Chemical synthesis

Compounds were synthesised from commercially available starting materials, and fully characterised by Infrared (IR) Spectroscopy, Mass Spectrometry (ESI-MS, HRMS-EI) and Nuclear Magnetic Resonance (^1^H, ^13^C NMR, HSQC, HMBC). Full experimental details and analytical data are provided in S1 Supporting Information.

### *In vivo* establishment of infection

100 infective embryonated eggs were incubated with water, water plus DMSO vehicle, or test compounds at a final concentration of 100μM for 14 days at room temperature. Eggs were then washed, re-suspended in water and 30 eggs given to a mouse via oral gavage. At day 15 post-infection, mice were culled and the number of worms present in the caecae and colon was counted. One data point was excluded: a water control individual mouse where there were technical difficulties with the oral gavage leading to the mouse regurgitating some of the *T*. *muris* eggs. It was ear marked at day of gavage and removed from the analysis.

We collected data using the same experimental design on different occasions, these are referred to as batches. Data were obtained from three batches (DMSO and water controls, batches [A,B,C]) or two batches (OX02983 [A,C], OX03153 [B,C]) with the total number of mice used, n, as follows: DMSO: 14, water: 16, OX02983: 10, OX03153: 12. The data were analysed by 2-way ANOVA (worms ~ treatment + batch + treatment * batch). There was no significant interaction effect (P = 0.14) or effect of batch (P = 0.47). We therefore combined the data from the three batches. A one-way ANOVA (worms ~ treatment) showed a significant difference between groups (F(3,48) = 8.3, P< 0.0005). We then conducted a post-hoc test using Tukey’s honest significant difference test.

### Embryonation assay

To assess whether the dihydrobenzoxazepinone compounds could also prevent embryonation, 100 un-embryonated eggs were treated with OX02983 and OX03153 at 100μM for 56 days in the dark at room temperature, then examined under an Olympus SD-ILK microscope for signs of embryonation.

### Cytotoxicity testing

CMT-93 murine rectal epithelial cells were maintained in Dulbecco's Modified Eagle's Medium (DMEM) with 10% fetal calf serum (FCS) and 1% penicillin-streptomycin solution (10,000U/ml). Cells were plated in 96-well tissue culture plates at a density of 2x10^2^ cells per well in a volume of 100μl, and cultured for 96 hours (37°C, 5% CO_2_) until reaching 30% confluence. The growth medium was removed and replaced with treatment media (DMEM, 1% FCS, 1% penicillin-streptomycin solution, 100μl assay volume) supplemented with vehicle alone (DMSO), test compounds or chlorpromazine, a positive control for cytotoxicity (final concentrations from 0 to 100μM). Maximum vehicle concentration for all compounds was 0.5% DMSO. Cells were cultured with the test compounds for 72 hours before cytotoxicity measurement.

The WST-8 assay was performed using the Cell Counting Kit– 8 (Sigma Aldrich # 96992). 10μl of the CCK-8 solution was added to each well, plates were incubated for a further 2 hours, before absorbance was measured at 450nm using a microplate reader. This assay measures the ability of metabolically active cells to reduce a water-soluble formazan dye.

Following the WST-8 assay, the medium was removed, cells were washed with 100μl PBS and then 100μl of neutral red medium (DMEM, 1% FBS and 33μg/ml neutral red dye (Sigma Aldrich #N2889) were added to each well, followed by incubation for a further 2 hours. The neutral red medium was removed, cells washed with 100μl of PBS and 100μl of destain solution (50% ethanol, 49% distilled water and 1% glacial acetic acid) were added. Each assay plate was shaken rapidly for 10 minutes before absorbance was measured at 540nm using a microplate reader. This assay measures the ability of living cells to actively take up the neutral red dye.

Results were analysed using GraphPad Prism and fitted using a log-logistic model.

### Compound property analysis and preliminary Structure-Activity Relationships (SAR)

Analyses of compound properties and structure-activity relationships were performed using DataWarrior [28] and *rcdk* [29].

## Results

### Screening for molecules that paralyse *ex vivo T*. *muris* adults

**Fig 2:**
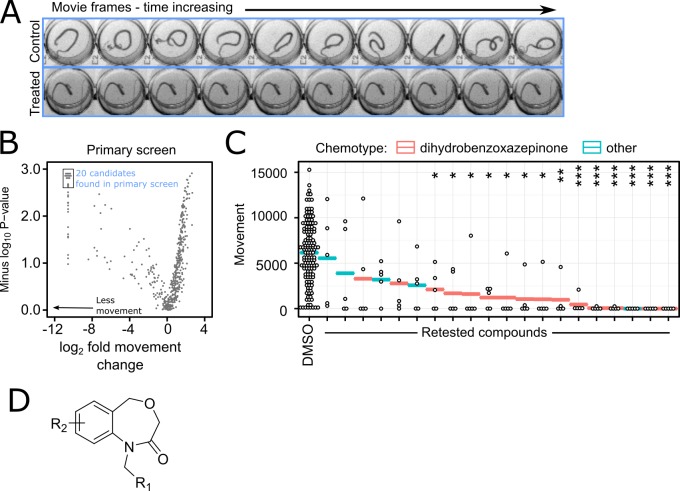
Hit compounds identified in a screen for molecules that paralyse whole *T*. *muris* parasites. (A) Movie frames showing complete worm paralysis. A montage of frames for control (top row wells) and hit compound treated (bottom row wells) is displayed; each frame is 4 seconds apart (B) Volcano plot of primary screen (n = 4, Mann-Whitney-Wilcoxon test, compounds tested at 100**μ**M) showing significant reduction in worm movement for 20 candidate compounds. (C) Of the 20 hit compounds from the primary screen that were selected for further study, 14 were active in the secondary screen (P < 0.05, Mann-Whitney-Wilcoxon test, corrected for multiple comparisons by Holm’s method. *** = P < 0.005, ** = P < 0.01, * = P < 0.05.) (D) 13 of the active compounds contain the same core dihydrobenzoxazepinone group. R_1_ = Aryl or Cycloalkyl. R_2_ = Aryl or Heteraryl.

The biology of *Trichuris* is comparatively poorly understood. In particular, as a clade I nematode it is only distantly related to the model nematode *Caenorhabditis elegans* [24], so we have limited information on potential targets for *Trichuris* control. We therefore decided to undertake a whole-organism phenotypic screen, searching for novel small molecules that paralyse or otherwise reduce the movement of adult *T*. *muris* parasites, screened *ex vivo*. Using an automated high-throughput motility analysis system, we screened 480 drug-like compounds from compound library DMSO solution samples in a pilot screen, with 4 biological replicates. 96 DMSO-only wells were used as negative controls. As shown in still frames selected from the raw movie data (Fig 2A), control worms swim vigorously in solution, but the most active compounds reduce movement to zero.

The results are summarised as a volcano plot (Fig 2B). Compounds that reduced movement and were highly reproducible (statistically significant) are shown in the top left-hand corner of this plot. To select hit compounds for future analysis we applied cut-offs of a movement reduction of at least 2^10^-fold in the automated motility assay and a significance value compared to the DMSO-only controls of P < 0.005 (Mann-Whitney test). 20 compounds were selected for further study. These compounds were blindly rescreened in the same adult motility assay (Fig 2C). 14/20 compounds were active in this assay (P < 0.05, Mann-Whitney test with Holm’s correction for multiple comparisons).

Interestingly, 13 of these active compounds from the screen contained the same core dihydrobenz[*e*][1,4]oxazepin-2(3*H)*-one structure (Fig 2D, hereafter referred to as dihydrobenzoxazepinones, or DHBs). No anthelmintic activity has previously been reported for this structural family and indeed it has not been implicated in any invertebrate control approach. Importantly, the DHB chemical class is both drug-like [30], and highly tractable for modification of the various structural motifs therein. This is critical as it provides a means to explore synthetically the prospect of optimising further both the intrinsic activity and the physicochemical and pharmacokinetic properties of the compound class.

Six compounds caused an increase in movement of at least 5-fold in the primary screen. The biological significance of this is not clear, although in the nematode *Caenorhabditis elegans*, treatments including dietary restriction lead to swimming hyperactivity [31]. However given our focus on anthelmintic discovery we focused on the compounds that reduced movement in our assay.

### Elaboration of the dihydrobenzoxazepinone active family

Critical to the development of small molecule drugs is the establishment of the relationship between structure and activity. This allows iterative design, testing and improvement of key characteristics that determine the success of a compound such as potency, toxicity and pharmacokinetics.

**Fig 3:**
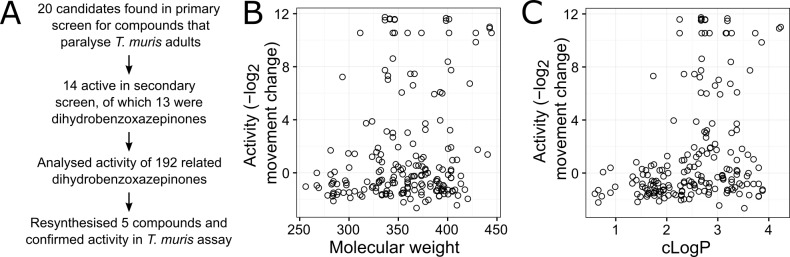
Elaboration of initial active compounds by characterising a diverse set of 192 dihydrobenzoxazepinones with the same core structure. (A) Screening and elaboration procedure. (B) Relationship between activity and molecular weight of each dihydrobenzoxazepinone. Activity is the minus log_2_ movement reduction compared to DMSO-only controls when assayed at 100**μ**M. (C) Relationship between activity and predicted hydrophobicity (cLogP) of each dihydrobenzoxazepinone.

We have undertaken a preliminary analysis by examining the activity in our primary assay of a set of 192 related dihydrobenzoxazepinones with the core structure shown in Fig 2D. Of these 20 are significantly active in the *T*. *muris* adult motility assay. Because the assay is fully automated and provides a movement measurement for each well, we have determined a quantitative activity estimate for all 192 dihydrobenzoxazepinones in this assay (Fig 3A), where activity is the minus log_2_ movement reduction compared to DMSO-only controls when assayed at 100μM. This allows us to relate structure to activity. There is little correlation between molecular weight and activity (Fig 3B, Spearman’s *ρ* = 0.14) but a stronger relationship between predicted hydrophobicity and activity (Fig 3C, Spearman’s *ρ* = 0.33). It has previously been reported that compounds with higher aLogP are more likely to accumulate within the nematode *C*. *elegans*, probably reflecting a greater ability to cross a permeability barrier within the nematode cuticle [32]. Therefore, the higher hydrophobicity of the active dihydrobenzoxazepinones may reflect greater compound access into the nematode parasite.

Complete screening results and structures of the analysed dihydrobenzoxazepinones are presented in the S1 Dataset.

### Resynthesis of hits

**Fig 4:**
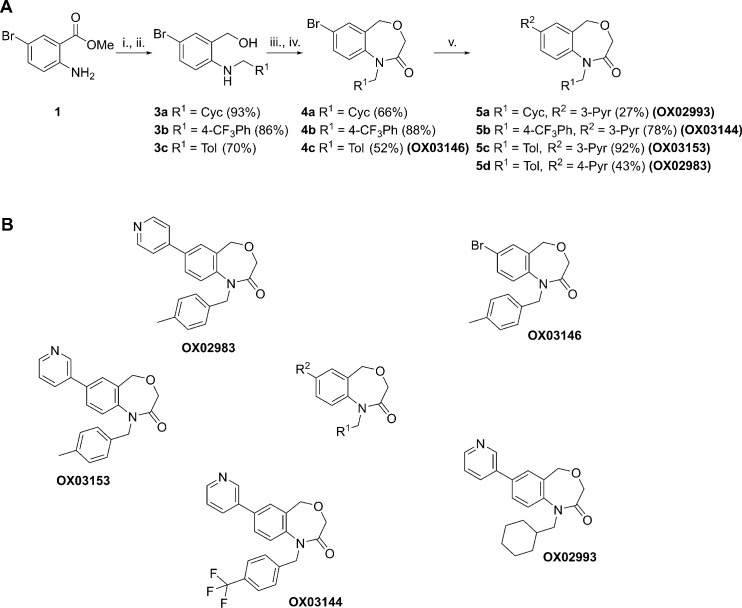
Synthetic route to putative hit compounds. (A) i. R^1^CHO (1.5 equiv.), AcOH (0.5 equiv.), NaBH(OAc)_3_, CH_2_Cl_2_, 0°C to RT, 2 days; ii. LiAlH_4_ (1M in THF, 3.5 equiv.); iii. chloroacetyl chloride (2.0–4.0 equiv.), NEt_3_ (2.0–4.4 equiv.), THF, 0°C to RT, 16 h; iv. 10N NaOH _(aq.)_, RT, 2 h; v. R^2^B(OH)_2_ (1.3 equiv.), Pd(PPh_3_)_4_ (5 mol%), 1.5M NaHCO_3 (aq.)_ (3 equiv.), DMF, 150 ^o^C (m.w.), 15 min. (B) Structures of resynthesized compounds.

Following any screening campaign it is routine to triage and confirm activity of putative active compounds by sourcing and retesting authentic, unambiguously characterised solid samples [33]. This is important since DMSO solution samples can degrade over time, and this often leads to so-called ‘false positive’ hits. We established a flexible and general synthetic route to this family of dihydrobenzoxazepinones (Fig 4A). Five members were resynthesized in the first instance (Fig 4B), using the protocol outlined in the reaction scheme (see S1 Supporting Information for further details). This modular route has the advantage of being able to provide access to a much wider set of compounds for a future optimisation programme. Details of the resynthesized compounds are given in Table 1.

**Table 1:** Structures of active dihydrobenzoxazepinones that were resynthesized, with selected calculated properties. RMM: relative molecular mass. #HBA: number of hydrogen bond acceptors. #HBD: number of hydrogen bond donors. tPSA: topological polar surface area. (Calculated using DataWarrior [28]).

Compound	PubChem SID	R_1_	R_2_	RMM	cLogP	#HBA	#HBD	tPSA (Å^2^)
**OX02983**	318018055	4-Tolyl	7-(Pyrid-4-yl)	344	3.1	3	0	42
**OX02993**	318018058	Cyclohexyl	7-(Pyrid-3-yl)	336	2.9	3	0	42
**OX03144**	318018059	4-CF_3_Ph	7-(Pyrid-3-yl)	398	3.6	6	0	42
**OX03146**	318018057	4-Tolyl	7-Br	346	3.6	2	0	30
**OX03153**	318018056	4-Tolyl	7-(Pyrid-3-yl)	344	3.1	3	0	42

### Resynthesized dihydrobenzoxazepinones show dose-dependent activity against *T*. *muris* parasites

**Fig 5:**
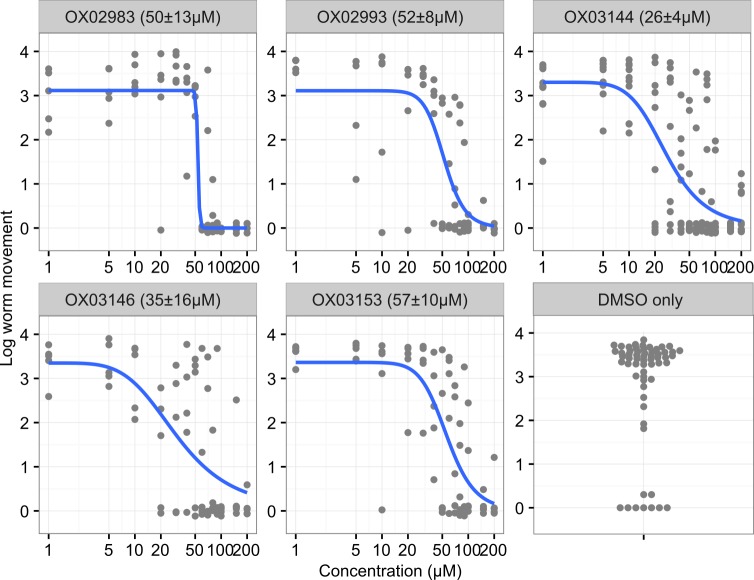
Resynthesized dihydrobenzoxazepinones show dose-dependent attenuation of *ex vivo T*. *muris* adult motility. Single adult worms were incubated for 24 hours in wells containing media plus compound. Motility was quantified using an automated phenotyping platform. EC_50_ ± standard error shown in parentheses. n = 5, except OX03144 where n = 10. Curve fitted using the four parameter log-logistic model.

Having resynthesized selected members of the active dihydrobenzoxazepinone class, we wanted to confirm their activity in the *ex vivo* adult *T*. *muris* motility assay. We tested each compound and determined the concentration-dependence of their activity between 1–200μM. These results are presented in Fig 5.

The EC_50_ values of these compounds, around 25–50μM, are acceptable considering this assay screens the whole parasite where compound access and compound metabolism will limit the engagement of the molecules with the target. Encouragingly these EC_50_ values are comparable with or exceed those for existing major anthelmintics in related *T*. *muris ex vivo* motility assays. Ivermectin is not active (maximum concentration tested 200μg/ml or 229μM) against *T*. *muris* L4 larvae [16] or against L1 larvae (maximum concentration tested 50μg/ml or 57μM) [14]. Mebendazole is not active against *T*. *muris* L1 larvae (maximum concentration tested 50μg/ml or 169μM) [14] but was active at 200μg/ml (678μM) though not 100μg/ml (339μM) against L3/L4 and adult *T*. *muris* [23]. Levamisole is active against *T*. *muris* adults in the automated assay utilised in this study (EC_50_ = 8 μM). Levamisole is effective at treating trichuriasis *in vivo*, although the single-dose cure rate, 9%, is low. This suggests that these early-stage dihydrobenzoxazepinone compounds already show promise for successful development as anti-*Trichuris* agents and supports future optimisation of structure and activity.

Structures and assay data for the five confirmed hit dihydrobenzoxazepinones have been submitted to the database PubChem (https://pubchem.ncbi.nlm.nih.gov/). Accession numbers for this data are shown in Table 1.

### Dihydrobenzoxazepinones are selective for the parasite with limited cytotoxicity to mouse gut epithelial cells

It was critical to ensure that our compounds showed selective activity against the parasite, as opposed to being generally toxic; as for example gut cytotoxicity may result in a limited therapeutic window. To address this, we assessed the resynthesized dihydrobenzoxazepinones for cytotoxicity in the mouse gut epithelial cell line CMT-93. We used two distinct assays, the WST-8 assay, which measures metabolic activity, and the neutral red accumulation assay, which measures the ability of living cells to actively take up the dye. Chlorpromazine was used as a positive control for cytotoxicity. The results of these experiments are shown in Table 2. Most of the dihydrobenzoxazepinones had no or weak cytotoxicity (EC_50_ > 100 μM). Interestingly, the most cytotoxic compound tested, OX03146, replaces the 7-pyridyl group with a smaller and more lipophilic 7-bromo substituent (Table 1). Along with the reduced polar surface area (tPSA), and likely improved membrane permeability [34], this might make the compound more promiscuous in binding to off-target proteins, thereby potentially explaining the increased cytotoxicity.

**Table 2:** Dihydrobenzoxazepinones cause limited cytotoxicity against mouse epithelial cell lines demonstrating selectivity for the pathogen target. Mouse CMT-93 rectal epithelial cells were used for this assay. Maximum tested concentration was 100**μ**M. n = 8, error range (in parentheses) shows 95% confidence interval. EC_50_ values in the adult *Trichuris* paralysis assay (Fig 5) are shown for comparison.

Compound	EC_50_ WST-8 assay (μM)	EC_50_ Neutral red assay (μM)	EC_50_ in adult *Trichuris* paralysis assay (μM)
Chlorpromazine (positive control)	9 (5–15)	10 (7–16)	n/a
OX02983	>100	>100	50
OX02993	>100	>100	52
OX03144	>100	60 (39–93)	26
OX03146	63 (37–111)	47 (31–71)	35
OX03153	>100	>100	57

Taken together, these data suggest that at this early stage, our dihydrobenzoxazepinone hit compounds show adequate selectivity for the parasite target versus the murine/human host. Improving this selectivity will be one of the goals of the continuing medicinal chemistry program to progress the optimisation and development of this family of compounds.

### Treatment of eggs with dihydrobenzoxazepinones reduces their ability to establish infection *in vivo*

**Fig 6:**
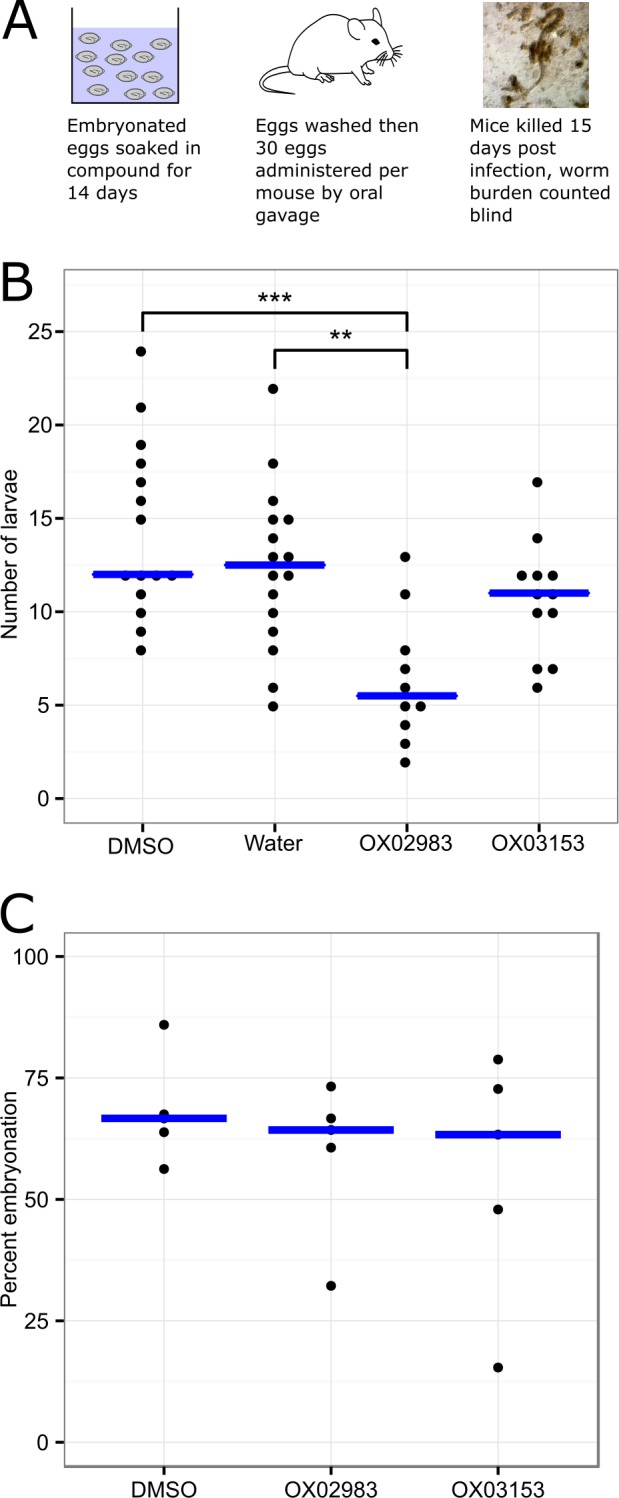
*T*. *muris* eggs treated with dihydrobenzoxazepinone OX02983 are less infective *in vivo*. (A) Experimental scheme: embryonated eggs were soaked in compound, and then used to infect mice by oral gavage. At day 15 post-infection, mice were culled and worm burden assessed. (B) Treatment with OX02983 reduced the ability of embryonated eggs to establish infection *in vivo*. Blue bar indicates median worm burden. A one-way ANOVA (worms ~ treatment) showed a significant difference between treatment groups (F(3,48) = 8.3, P< 0.0005). Differences between groups were determined using a post-hoc Tukey HSD test (** = P<0.005, *** = P<0.0001). n = 14 (DMSO), 16 (water), 10 (OX02983), 12 (OX03153). (C) Dihydrobenzoxazepinones do not act by blocking embryonation. Eggs were incubated with 100μM compounds or DMSO-alone control for 56 days. Embryonation was then quantified. No significant differences between groups were detected: one-way ANOVA (embryonation ~ treatment, F(2,12) = 0.60, P = 0.57). Blue bar indicates median percentage embryonation.

Developing a novel pharmaceutical for a neglected tropical disease such as trichuriasis is an economic and practical challenge. We wanted to consider whether an alternative strategy could be used to establish control of infection but with a potentially faster development process. As described in Fig 1, the lifecycle of both *T*. *muris* and *T*. *trichiura* involves a stage where unembryonated, uninfective eggs are released into the environment in the faeces. These eggs embryonate to become infective over a period of time and are then transmitted to further individuals via consumption of contaminated food or water. Therefore, interruption of the lifecycle by targeting of eggs in the environment is a potential alternative strategy to mass pharmaceutical treatment. We wanted to determine whether our compounds, when applied to eggs as if via an environmental treatment, could affect two key processes: establishment of infection and embryonation. We tested the first possibility by establishing an assay where embryonated *T*. *muris* eggs are soaked in the test compound, washed, and then applied to mice via oral gavage. Infectivity was quantified by culling the mice on day 15, and counting the number of worms that were hatched and able to establish infection in the mouse gut (Fig 6A). The results are summarised in Fig 6B. Dihydrobenzoxazepinone OX02983 was able to reduce infectivity *in vivo* (Tukey’s honest significant difference test, P < 0.005), by an estimated 54% (median DMSO control = 12.0 worms establishing infection, median OX02983 treated group = 5.5 worms). OX03153 was not active in this assay.

In contrast, the compounds were not able to block the earlier process of embryonation, where *T*. *muris* develops from a ball of cells into vermiform stage within the eggshell. This may reflect poor permeability of the compounds into the eggs, or indicate that they act later in development or interfere with the process of hatching or infection itself (Fig 6C).

These results support the potential use of dihydrobenzoxazepinones as a spray or other treatment that reduces the infectivity of *Trichuris* eggs within the environment, in particular at hotspots such as latrines. Such a use would however require improved potency to be achieved during future drug development.

## Discussion

### Current lack of effective treatments for trichuriasis

There are several distinct modes of action of current anthelmintics. Examples include benzimidazoles such as albendazole and mebendazole, which act by inhibiting microtubule synthesis [7], oxamniquine, which prevents nucleic acid synthesis [35], praziquantel, which is thought to act by increasing the permeability of schistosome cell membranes to calcium ions during infection [36], ivermectin inhibits parasite neurotransmission mainly via actions on L-glutamate gated chloride channels [37–40] and levamisole, oxantel and pyrantel are nicotinic receptor agonists [41]. However the limitations of current anthelmintic treatments are particularly pronounced in the context of regimes targeting *Trichuris*.

Mass Drug Administration (MDA) programs that aim to reduce and potentially eliminate soil-transmitted helminth infection in humans have predominantly relied on benzimidazole drugs alone, making this method of treatment vulnerable to the development of anthelmintic resistance [42]. Furthermore, single doses of benzimidazoles lead to low cure rates for infection with *T*. *trichiura*, only 28% and 36% for albendazole and mebendazole respectively [8]. Because of these low cure rates, modelling studies have concluded that MDA with benzimidazoles is unable to interrupt transmission of *T*. *trichiura* and thus achieve elimination in many settings [9].

Attempts have been made to boost the low single-dose cure rates of the benzimidazoles by combining them with other drugs. Studies conducted in school-aged children on Pemba Island, Tanzania, combined albendazole with a second anti-protozoal drug, nitazoxanide, which interferes with parasite anaerobic energy metabolism [43]. This combination treatment showed low cure rates against *T*. *trichiura* [44]. Similarly, drug trials conducted in Tanzania using combined treatments of albendazole plus ivermectin, show significant improvement in cure rates compared to albendazole or mebendazole alone, but these cure rates are also low (27.5%). Albendazole plus mebendazole was also used in the same study but also show low efficacy, as only 8.4% of test participants were cured [10]. Further multi-drug trials on Pemba Island Tanzania involving treatment with oxantel pamoate plus albendazole have resulted in higher cure (31% in one study and 68.5% in a second) and egg-reduction rates for *T*. *trichiura* infection when compared with standard therapy, however mild adverse effects were also reported [10,11]. These multi-anthelmintic drug treatments provide improved cure rates to *T*. *trichiura* infection, when compared to the use of single anthelmintics alone, but risk unwanted side effects and are not appropriate for MDA. Recently a dose-ranging study measured the effectiveness of oxantel pamoate alone against *T*. *trichiura* in children and found, at the optimum dose, a cure rate of 60% with a good tolerability profile [12]. This drug is clearly the most promising current alternative to the benzimidazoles. However it is not a panacea with limited cure rates [45], still low compared to anti-hookworm MDA. Modelling also suggests that even improved cure rates with drug-combination MDA are insufficient to achieve elimination in higher transmission settings [9].

Despite the clear need for alternative anthelmintic drugs to achieve control or ideally eradication of diseases such as trichuriasis, the pipeline for development of such drugs is dry [11]. A particularly important reason for the poor efficacy of existing drugs for trichuriasis is that they were developed primarily for animal health indications, for the control of major agricultural pests such as *Haemonchus contortus*. *Trichuris*, being a clade I nematode is evolutionarily distant from most of these economically important parasites, so drug targets for the control of this species are likely to be divergent. We therefore decided to initiate a program of drug discovery that was targeted to *Trichuris* from the very start by screening for loss of motility in *T*. *muris* adults.

### Dihydrobenzoxazepinones, a new class of anthelmintics active on *T*. *muris*

Following a pilot high-throughput screen, subsequent hit triage and confirmatory resynthesis, we have identified the dihydrobenzoxazepinones as a new class of drug-like molecules with activity on the model mouse parasite, *T*. *muris*. EC_50_ values of around 25–50μM for these early-stage, small molecules, activity data are comparable with the existing anthelmintic levamisole in the same assays. Encouragingly, cytotoxicity testing on murine gut epithelial cells showed that most compounds in this series had limited cytotoxicity, with EC_50_ values > 100μM, the maximum concentration tested. Further toxicity testing in diverse *in vitro* and *in vivo* systems will be required during the development of this compound series. However these data indicate that the dihydrobenzoxazepinone (DHB) class have selectivity for the parasite, and are encouraging in the context of optimising this series further for post-infection treatment, which will require strict safety and host tolerance criteria.

Importantly, the DHB chemical class is both drug-like [30], and highly tractable for modification of the various structural motifs therein. New analogues can be designed to improve the broader molecular properties of the class, this being critical for modification of key parameters such as solubility, permeability and metabolism–all of which are essential for development of a new therapeutic agent. It is important to note that the term ‘drug-like’ as employed in the contemporary medicinal chemistry literature is usually directed towards orally delivered systemic agents which are targeting a central or peripherally located target enzyme or receptor. For our compounds, which are targeting the gastrointestinal located *Trichuris*, it would be desirable to have compounds with relatively *minimal* systemic exposure, and therefore the conventional ‘drug-like’ parameters which affect solubility and permeability, including lipophilicity need not adhere to the ‘normal’ Lipinski type values [46]. In addition, a relative metabolic lability may be desirable, such that any systemically absorbed compound would be rapidly cleared.

The desirable absorption and distribution properties of an anti-*Trichuris* agent are complicated by the burrowing of the whipworm into intestinal epithelial cells, which may provide some protection from drugs. There is limited understanding of this aspect of *Trichuris* biology, but a study has determined, after oral treatment of pigs infected with whipworm *T*. *suis*, the concentration of various benzimidazole drugs in host plasma and absorbed into the parasite [47]. The tight relationship between plasma and worm concentrations of oxfendazole led to the conclusion that this drug reaches the worm from the circulation via intestinal cells, whereas the limited relationship between plasma and worm concentrations of febendazole indicated direct absorption from the intestinal lumen. The role of the bacillary band, a specialised epidermal structure, in drug uptake is also thought to be important [48]. Quantifying the absorption route and kinetics of the DHB compounds will be required to generate a pharmacokinetic model and therefore critical to the further development of this series as an anti-*Trichuris* agent.

No anthelmintic activity has previously been reported for the DHB structural family and indeed it has not been implicated in any invertebrate control approach. Although we have not as yet identified the molecular target for this class of molecules, the dihydrobenzoxazepinones are a novel family of anthelmintics specifically identified for their activity on *Trichuris*.

### Developmental route to a new oral drug for trichuriasis

The developmental process leading to the identification of a new drug is long, expensive, and prone to failure. The hit to lead stage of this process takes molecules identified in high-throughput screens and iteratively improves their activity in disease-relevant assays as well as building in properties important for drug action, particularly related to drug metabolism and pharmacokinetics (DMPK). Recent experience in drug development of neglected diseases has led to the establishment of criteria for the hit-to-lead process [49,50]. The DHB compounds meet all of the criteria for hit selection for infectious diseases of the developing world, such as confirmed activity with resynthesized compound, sigmoidal dose-dependence, tractable chemotype that passes drug-like filters, and a selectivity window for cytotoxicity in a mammalian cell line [49]. These compounds also meet some (but not all) of the criteria for lead compounds, such as establishment of a tractable route for synthesis that is amenable to rapid and diverse series expansion, and suitable drug-likeness. The *in vitro* activity of the compounds in motility assays (26–57μM, equivalent to 10–20μg/ml) also reaches the lead compound criterion (10μg/ml) for motility inhibition against the related microfilarial nematode *Brugia malayi* [50]. The largest gap in our understanding of the DHB compounds is related to DMPK. As discussed in the previous section, understanding these processes are critical to the development of orally active small molecules. If the DMPK properties of the DHB compounds can be determined and where necessary improved, it is possible that this chemotype will achieve the final milestone for lead compounds against helminth disease, namely a substantial reduction in worm burden in *in vivo* tests.

### Reduction of infectivity of eggs offers prospect of environmental treatment for trichuriasis

An exciting and complementary use of the novel anthelmintics is to use them in the environment to decrease the risk of infection by targeting the egg stage of the *T*. *trichiura* life cycle. This approach is particularly attractive as *T*. *trichiura* eggs are highly resistant to extreme temperature changes and ultraviolet radiation and thus remain viable in the environment for many years [51]. Dihydrobenzoxazepinone OX02983 was found to significantly reduce infectivity of eggs *in vivo* compared to eggs that had been treated under the same conditions with water or DMSO alone (Fig 6B). In contrast, there were no differences between the percentage of embryonation seen between dihydrobenzoxazepinone treated eggs and eggs treated with water or DMSO alone. This suggests that none of the active compounds were able to prevent embryonic development.

Further investigation is required to identify the mode of action of this compound on embryonated eggs. For example, the compound may reduce L1 viability within the egg, or alter the eggshell structure, preventing larval hatching inside the host. Chemical control of egg hatching would be particularly beneficial in breaking the *T*. *trichiura* life cycle in the field before the parasite can infect or re-infect the host. This mechanism of action would require environmentally stable and non-toxic compositions.

We envisage that, if an increase in potency is achieved during the drug development process, dihydrobenzoxazepinones could potentially be developed into a therapeutic agent that acts in the environment, perhaps via a spray utilised at sites of high-parasite density such as communal latrines, to interrupt the parasite lifecycle. This could allow for wider deployment in environments where mass drug administration is challenging. Consideration of environmental toxicity would be critical in this context. A further possibility is that mass drug administration with a compound with both anti-worm and anti-egg infectivity activity might, in addition to worm killing/expulsion, achieve a reduction of egg infectivity in the faeces, helping to break the parasite lifecycle.

### Possible animal health applications

This study was initiated based on the urgent need for new treatments for the neglected human disease trichuriasis. However the economic realities of the cost of drug development may mean that the financial impetus behind the development of new drugs for *T*. *trichiura* comes from the animal health industry via control of *Trichuris* species that infect agricultural and companion animals. The related species *Trichuris suis* and *Trichuris vulpis* infect pigs and dogs respectively, with *T*. *suis* infection causing economically-costly reduction in pig weight-gain [52–54]. Interestingly increasing outdoor rearing of pigs due to animal welfare considerations has resulted in accumulation of long-lived *T*. *suis* eggs in the soil, causing difficulties with controlling infection [52]. Therefore an environmental egg-targeted treatment that interrupts the parasite lifecycle may be particularly useful in this context.

## Conclusions

This study has identified a new family of molecules active against the important parasite *Trichuris*. We have found 20 active family members, from a total of 192 dihydrobenzoxazepinones tested, the activity being confirmed through resynthesis. This has allowed us to start to understand the molecular features that are important for activity against the parasite. The dihydrobenzoxazepinone compounds that we have identified are drug-like and ideal for further iterative optimisation and improvement as part of the drug development process. One member of the dihydrobenzoxazepinone class, OX02983, was also effective at reducing the ability of eggs to establish infection *in vivo*, pointing the way to a potential environmental treatment for trichuriasis. The successful identification of anthelmintic compounds using our automated motility phenotyping platform supports the utility of this approach, and could potentially be used with a variety of animal and human nematodes and other parasites.

## Supporting information

S1 Supporting Information Methodological detail and supporting spectra for the resynthesized dihydrobenzoxazepinone compounds. (DOCX)

S1 Dataset File containing structure and assay information on screened dihydrobenzoxazepinones. (XLSX)
