# Supplementary material for: Dihydrobenz[e][1,4]oxazepin-2(3H)-ones, a new anthelmintic chemotype immobilising whipworm and reducing infectivity in vivo
Source: PLoS Negl Trop Dis. 2017 Feb 9;11(2):e0005359. doi: 10.1371/journal.pntd.0005359 (PMC5321434; doi:10.1371/journal.pntd.0005359)
Supplement: S1 Supporting Information — (DOCX) [file pntd.0005359.s001.docx]

**General Experimental**

Thin layer chromatography (TLC) was performed on aluminium sheets coated with 60 F_254_ silica. NMR spectra were recorded on Bruker AV400 (400 MHz) or Bruker AVII 500 (500 MHz) instruments in the deuterated solvent stated. All chemical shifts (δ) are quoted in ppm and coupling constants (*J*) in Hz. Residual signals from the solvents were used as an internal reference using the stated deuterated solvent. Infrared spectra were recorded on a Perkin-Elmer 1750 IR Fourier Transform spectrophotometer using thin films on a diamond ATR surface (thin film). Only the characteristic peaks are quoted. Melting points were determined using a Stanford Research Systems EZ-Melt. Low resolution mass spectra (*m/z*) were recorded on an Agilent 6120 spectrometer and high resolution mass spectra (HRMS *m/z*) on a Bruker microTOF mass analyzer using electrospray ionization (ESI) or field ionization (FI). Reactions with microwave irradiation were carried out in a Biotage Initiator microwave synthesizer. Petroleum refers to the fraction boiling between 30-40 ^o^C.

i. R^1^CHO (1.5 equiv.), AcOH (0.5 equiv.), NaBH(OAc)_3_, CH_2_Cl_2_, 0 ^o^C to RT, 2 days; ii. LiAlH_4_ (1M in THF, 3.5 equiv.); iii. chloroacetyl chloride (2.0-4.0 equiv.), NEt_3_ (2.0-4.4 equiv.), THF, 0 ^o^C to RT, 16 h; iv. 10N NaOH _(aq.)_, RT, 2 h; v. R^2^B(OH)_2_ (1.3 equiv.), Pd(PPh_3_)_4_ (5 mol%), 1.5M NaHCO_3_ _(aq.)_ (3 equiv.), DMF, 150 ^o^C (m.w.), 15 min.

# General Procedure 1.

Desired aniline (1.0 equiv.) was dissolved in anhydrous CH_2_Cl_2_ (6 mL/mmol) at RT, before addition of the corresponding aldehyde (1.5 equiv.) and AcOH (0.5 equiv.). The solution was then cooled to 0°C (external) before addition of NaBH(OAc)_3_ (2.0 equiv.). The reaction mixture was allowed to warm slowly to RT and the mixture was stirred at that temperature for 2 days. The reaction was diluted with further CH_2_Cl_2_ (5 mL/mmol), quenched with sat. aq. NaHCO_3_ (10 mL/mmol), and the aqueous layer was further extracted with CH_2_Cl_2_ (5 x 10 mL/mmol), the combined organic layers were dried (Na_2_SO_4_) and concentrated *in vacuo*. The compound was then further purified by flash column chromatography (silica gel).

# Methyl 5-bromo-2-((cyclohexylmethyl)amino)benzoate (2a)

Following general procedure 1, **2a** was obtained from methyl-2-amino-5-bromobenzoate (200 mg, 0.87 mmol, 1.0 equiv.) and cyclohexanecarboxaldehyde (0.16 mL, 1.305 mmol, 1.5 equiv.). Purification by flash column chromatography (EtOAc: Petroleum; 1:19) afforded the title compound as a pale yellow solid (262mg, 93%).

mp = 46-47 ^o^C; R_f_ = 0.67 (EtOAc: Petroleum; 1:19); ν_max_ (film)/cm^-1^= 3349w (NH), 2919m (CH), 2849w (CH), 1687m (C=O), 1574m (CO−O), 1501m (C=C_arom_), 1435m (C=C_arom_), 1206s (C−O); ^1^H NMR (500 MHz, CDCl_3_) δ 7.98 (1H, d, ^4^*J*_HH_ = 2.5 Hz), 7.78 (1H, t, ^3^*J*_HH_ = 5.1 Hz), 7.38 (1H, dd, ^3^*J*_HH_ = 9.1, ^4^*J*_HH_ = 2.5 Hz), 6.55 (1H, d, ^3^*J*_HH_ = 9.1 Hz), 3.85 (3H, s), 3.00 (2H, dd, ^3^*J*_HH_ = 6.8 Hz, ^3^*J*_HH_ = 5.1 Hz), 1.87-1.80 (2H, m), 1.78-1.72 (2H, m), 1.70-1.60 (2H, m), 1.30-1.15 (3H, m), 1.05-0.97 (2H, m); ^13^C NMR (126 MHz, CDCl_3_) δ 168.3, 150.5, 137.3, 133.9, 113.3, 111.0, 105.4, 51.8, 49.7, 37.5, 31.4, 26.6, 26.1; LRMS (ESI^+^) calculated for [C_15_H_21_^79^BrNO_2_+H]^+^ = 326.1, found 326.1, [M+H]^+^, 100%, calculated for [C_15_H_21_^81^BrNO_2_+H]^+^ = 328.1, found 328.1, [M+H]^+^, 90%; HRMS (ESI^+^) calculated for [C_15_H_21_^79^BrNO_2_+H]^+^ = 326.0750, found 326.0751, [M+H]^+^, calculated for [C_15_H_21_^81^BrNO_2_+H]^+^ = 328.0730, found 328.0730, [M+H]^+^.

# Methyl 5-bromo-2-((4-(trifluoromethyl)benzyl)amino)benzoate (2b)

Following general procedure 1, **2b** was obtained from methyl-2-amino-5-bromobenzoate (200 mg, 0.87 mmol, 1.0 equiv.) and 4‑(trifluoromethyl)-benzaldehyde (0.1 8mL, 1.31 mmol, 1.5 equiv.). Purification by flash column chromatography (EtOAc:Petroleum; 1:19) afforded the title compound as a yellow solid (302 mg, 93%).

mp = 76-77 ^o^C; R_f_ = 0.56 (EtOAc:Petroleum; 1:19); ν_max_ (film)/cm^-1^= 3376m (NH), 3309w (CH), 2956w (CH), 1682m (C=O), 1574m (CO−O), 1505m (C=C_arom_), 1437m (C=C_arom_);^1^H NMR (500 MHz, CDCl_3_) δ 8.26 (1H, t, ^3^*J*_HH_ = 5.0 Hz) 8.04 (1H, d, ^4^*J*_HH_ = 2.5 Hz), 7.59 (2H, d, ^3^*J*_HH_ = 7.9 Hz) 7.43 (2H, d, ^3^*J*_HH_ = 7.9 Hz), 7.34 (1H, dd, ^3^*J*_HH_ = 9.0 Hz, ^4^*J*_HH_ = 2.5 Hz), 6.43 (1H, d, ^3^*J*_HH_ = 9.0 Hz), 4.50 (2H, d, ^3^*J*_HH_ = 5.8 Hz), 3.88 (3H, s); ^13^C NMR (126 MHz, CDCl_3_) δ 168.2, 149.6, 142.7, 137.4, 134.1, 129.8 (q, ^2^*J*_CF_ = 32.4 Hz, C1’) 127.3, 125.9 (q, ^3^*J*_CF_ = 3.8 Hz) 124.3 (q, ^1^*J*_CF_ = 271.8 Hz) 113.6, 112.1, 106.9, 52.0, 46.7; LRMS (ESI^+^) calculated for [C_16_H_13_^79^BrF_3_NO_2_+H]^+^ = 388.0, found 388.0, [M+H]^+^, 95%, calculated for [C_16_H_13_^81^BrF_3_NO_2_+H]^+^ = 390.0, found 390.0, [M+H]^+^, 100%; HRMS (ESI^+^) calculated for [C_16_H_13_^79^BrF_3_NO_2_+H]^+^ = 388.0155, found 388.0153, [M+H]^+^, calculated for [C_16_H_13_^81^BrF_3_NO_2_+H]^+^ = 390.0134, found 390.0133, [M+H]^+^.

# Methyl 5-bromo-2-((4-methylbenzyl)amino)benzoate (2c)

Following general procedure 1, **2c** was obtained from was obtained from methyl-2-amino-5-bromobenzoate (200 mg, 0.87 mmol, 1.0 equiv.) and *p*‑tolualdehyde (0.15 mL, 1.31 mmol, 1.5 equiv.). Purification by flash column chromatography (EtOAc:Petroleum; 1:19) afforded the title compound as a pale yellow solid (202 mg, 70%).

mp = 70-72 ^o^C; *R*_f_ =0.60 (EtOAc: Petroleum); ν_max_ (film)/cm^-1^= 3357w (NH), 2955w (CH), 2851w (CH), 1688s (C=O), 1571m (CO−O), 1499m (C=C_arom_), 1434m (C=C_arom_), 1365m (C−O), 1309s (C‑O); ^1^H NMR (500 MHz, CDCl_3_) δ 8.13 (1H, t, ^3^*J*_HH_ = 4.9 Hz), 8.02 (1H, d, ^4^*J*_HH_ = 2.6 Hz), 7.34 (1H, dd, ^3^*J*_HH_ = 9.2 Hz, ^4^*J*_HH_ = 2.6 Hz), 7.22 (2H, d, ^3^*J*_HH_ = 8.1 Hz), 7.14 (2H, d, ^3^*J*_HH_ = 8.1 Hz), 6.52 (1H, d, ^3^*J*_HH_ = 9.2 Hz), 4.38 (2H, d, ^3^*J*_HH_ = 5.5 Hz), 3.86 (3H, s), 2.34 (3H, s); ^13^C NMR (126 MHz, CDCl_3_) δ 168.2, 150.0, 137.3, 137.1, 135.3, 133.9, 129.6, 127.1, 113.7, 111.7, 106.2, 51.9, 46.9, 21.2; LRMS (ESI^+^) calculated for [C_16_H_16_^79^BrNO_2_+H]^+^ = 334.0, found 334.0 [M+H]^+^ , 100%, calculated for [C_16_H_16_^81^BrNO_2_+H]^+^ = 336.0, found 336.0 [M+H]^+^, 70%; HRMS (ESI^+^) calculated for [C_16_H_16_^79^BrNO_2_+H]^+^ = 334.0437, found 334.0437, calculated for [C_16_H_16_^81^BrNO_2_+H]^+^ = 336.0417, found 334.0416.

**General Procedure 2.**

Desired methyl ester (1.0 equiv.) was dissolved in anhydrous THF (7 mL/mmol) under a N_2_ atmosphere and cooled to 0 °C (external). LiAlH_4_ (1.0 M in THF, 3.5 equiv.) was then added dropwise over a period of 5-10 min and the reaction mixture was stirred at that temperature for 1 h. Sat. aq. NH_4_Cl (3 mL/mmol) was then added and the aqueous layer extracted with EtOAc (3 x 35mL/mmol), the combined organic layers were then dried (Na_2_SO_4_) and concentrated *in vacuo*. The reaction product was then carried forward without the requirement for further purification.

**5-Bromo-2-((cyclohexylmethyl)amino)benzyl alcohol (3a)**

Following general procedure 2, **3a** was obtained from **2a** (2.0 g, 6.1 mmol, 1.0 equiv.) and LiAlH_4_ (1M in THF, 21.4 mL, 21.4 mmol, 3.5 equiv.). Purification by flash column chromatography (EtOAc:Petroleum; 3:17) afforded the compound as a clear colourless oil, (1.8 g, quant.).

R_f_ = 0.70 (EtOAc:Petroleum; 3:17); ν_max_ (film)/cm^-1^= 3402br (OH), 3311w (NH), 2952m (CH), 2847m (CH), 1505m (C=C_arom_), 1444m (C=C_arom_); ^1^H NMR (500 MHz, CDCl_3_) δ 7.27 (1H, dd, ^3^*J*_HH_ = 8.7 Hz, ^4^*J*_HH_ = 2.4 Hz), 7.15 (1H, d, ^4^*J*_HH_ = 2.4 Hz), 6.51 (1H, d, ^3^*J*_HH_ = 8.7 Hz), 4.60 (2H, s), 2.94 (2H, d, ^3^*J*_HH_ = 6.6 Hz), 1.85-1.56 (6H, m), 1.32-1.13 (3H, m), 1.04-0.94 (2H, m); ^13^C NMR (126 MHz, CDCl_3_) δ 147.1, 132.2, 131.6, 125.9, 112.2, 107.4, 64.5, 50.3, 37.4, 31.5, 26.7, 26.1; LRMS (ESI^+^) calculated for [C_14_H_20_^79^BrNO+H]^+^ = 298.1, found 298.1 [M+H]^+^ 100%, calculated for [C_14_H_20_^81^BrNO+H]^+^ = 300.1, found 300.1 [M+H]^+^ 100%; HRMS (ESI^+^) calculated for [C_14_H_20_^79^BrNO+H]^+^ = 298.0801, found [M+H]^+^ 298.0803, calculated for [C_14_H_20_^81^BrNO+H]^+^ = 300.0781, found 300.0780 [M+H]^+^.

**Methyl 5-bromo-2-((4-(trifluoromethyl)benzyl)amino)benzyl alcohol (3b)**

Following general procedure 2, **3b** was obtained from **2b** (1.3 g, 3.4 mmol, 1.0 equiv.) and LiAlH_4_ (1M in THF, 11.9 mL, 11.9 mmol, 3.5 equiv.). Purification by flash column chromatography (EtOAc:Petroleum; 1:4) afforded the compound as an off-white solid (985 mg, 92%).

mp = 79-82^o^C; R_f_ = 0.38 (EtOAc:Petroleum; 1:4); ν_max_ (film)/cm^-1^= 3311br (OH and NH), 2921w (CH), 1444m (C=C_arom_), 1419m (C=C_arom_); ^1^H NMR (400 MHz, CDCl_3_) δ 7.59 (2H, d, ^3^*J*_HH_ = 8.4 Hz), 7.46 (2H, d, ^3^*J*_HH_ = 8.4 Hz), 7.24-7.19 (2H, m), 6.40 (1H, d, ^3^*J*_HH_ = 8.5 Hz), 5.40 (1H, s), 4.68 (2H, s), 4.44 (2H, s); ^13^C NMR (101 MHz, CDCl_3_) δ 146.1, 143.3, 132.2, 131.7, 129.6 (q, ^2^*J*_CF_ = 31.6 Hz), 127.4, 126.4, 125.8 (q, ^3^*J*_CF_ = 3.8 Hz), 124.3 (q, ^1^*J*_CF_ = 271.8 Hz), 112.7, 108.8, 64.3, 47.3; LRMS (ESI^+^) calculated for [C_15_H_13_^79^BrF_3_NO+H]^+^ 360.0, found 360.0, [M+H]^+^, 99%, calculated for [C_15_H_13_^81^BrF_3_NO+H]^+^ = 362.0, found 362.0 [M+H]^+^, 100%; HRMS (ESI^+^) calculated for [C_15_H_13_^79^BrF_3_NO+H]^+^ = 360.0205, found 360.0208, [M+H]^+^, calculated for [C_15_H_13_^81^BrF_3_NO+H]^+^ = 362.0185, found 362.0187 [M+H]^+^.

**5-Bromo-2-((4-methylbenzyl)amino)benzoate (3c)**

Following general procedure 2, **3c** was obtained from **2c** (1.0 g, 3.0 mmol, 1.0 equiv.) and LiAlH_4_ (1M in THF, 10.5 mL, 10.5 mmol, 3.5 equiv.). Purification by flash column chromatography (EtOAc:Petroleum; 1:4) afforded the compound as a clear colourless oil (0.9 g, 98%).

R*_f_* = 0.60; (EtOAc: Petroleum; 1:4); ν_max_ (film)/cm^-1^= 3414m (NH), 3339br (OH), 2921w (CH), 2872w (CH), 15 10m (C=C_arom_), 1478m (C=C_arom_), 1452m (C=C_arom_); ^1^H NMR (500 MHz, CDCl_3_) δ 7.28-7.23 (3H, m), 7.18 (1H, d, ^3^*J*_HH_ = 2.3 Hz), 7.15 (2H, d, ^3^*J*_HH_ = 8.1 Hz), 6.51 (1H, d, ^3^*J*_HH_ = 8.6 Hz), 4.64 (2H, s), 4.31 (2H, s), 2.34 (3H, s); ^13^C NMR (500 MHz, CDCl_3_) δ 146.5, 137.1, 135.9, 132.2, 131.6, 129.5, 127.4, 126.3, 112.8, 108.3, 64.3, 47.6, 21.3; LRMS (ESI^+^) calculated for [C_15_H_16_^79^BrNO+H]^+^ = 306.1, found 306.0 ([M+H]^+^ 100), calculated for = 308.1 [C_15_H_16_^81^BrNO+H]^+^ found 308.0 ([M+H]^+^ 100); HRMS (ESI^+^), calculated for [C_15_H_16_ON^79^Br+H]^+^ = 306.0488, found [M+H]^+^ 306.0490, calculated for [C_15_H_16_ON^81^Br+H]^+^ = 308.0468, found [M+H]^+^ 308.0468.

**General Procedure 3.**

To a solution of desired alcohol in NEt_3_ in anhydrous THF (2 mL/mmol) at 0 °C (external) was slowly added chloroacetyl chloride. The solution was allowed to slowly warm to RT overnight, at which time the crude reaction mixture was passed through a short pad of silica gel, washing with EtOAc (ca 15ml/mmol) and the volatiles were removed under reduced pressure. The crude residue was then dissolved in *^i^*PrOH (2 mL/mmol) before addition of 10N NaOH. The solution was stirred at RT for 2h, diluted with CH_2_Cl_2_ (ca 5 mL/mmol), washed with sat. aq. brine (ca 5mL/mmol) and then further extracted with CH_2_Cl_2_ (ca 5mL/mmol). The combined organic layers were dried (Na_2_SO_4_) and then concentrated *in vacuo*. The compound was then further purified by flash column chromatography (silica gel).

**7-Bromo-1-(cyclohexylmethyl)-1,5-dihydrobenzo[e][1,4]oxazepin-2(3H)-one (4a)**

Following general procedure 3, **4a** was obtained from **3a** (0.50 g, 1.7 mmol, 1.0 equiv.), chloroacetyl chloride (0.53 mL, 6.7 mmol, 4.0 equiv.) and NEt_3_ (1.0 mL, 7.4 mmol, 4.4 equiv.) for the acylation, followed by subsequent ester hydrolysis and concomitant cyclisation using aq. 10N NaOH (0.37 mL, 3.7 mmol, 2.2 equiv.). Purification by flash column chromatography (EtOAc:Petroleum; 3:17) afforded the compound as am off-white solid (0.38g, 66%).

mp = 109-110 ^o^C; ν_max_ (film)/cm^-1^= 2923w (CH), 2858w (CH), 1654s (C=O), 1484m (C=C_arom_), 1420m (C=C_arom_); R*_f_* = 0.20 (EtOAc:Petroleum; 3:17); ^1^H NMR (500 MHz, CDCl_3_) δ 7.57 (1H, dd, ^3^*J*_HH_ = 8.7 Hz, ^4^*J*_HH_ = 2.3 Hz), 7.51 (1H, d, ^3^*J*_HH_ = 2.3 Hz), 7.15 (1H, d, ^3^*J*_HH_ = 8.7), 4.62, (2H, s), 3.97 (2H, s), 3.78 (2H, d, ^3^*J*_HH_ = 6.8), 1.72-1.59 (6H, m), 1.19-1.09 (3H, m), 1.00-0.89 (2H, m); ^13^C NMR (500 MHz, CDCl_3_) δ 168.3, 142.1, 133.7, 133.2, 131.3, 122.8, 119.3, 68.0, 67.7, 52.8, 36.8, 31.3, 26.3, 25.8; LRMS (ESI^+^) calculated for [C_16_H_20_^79^BrNO_2_+H]^+^ = 338.1, found 338.0 [M+H]^+^, 50%, calculated for [C_16_H_20_^79^BrNO_2_+Na]^+^ = 360.1, found 360.1 [M+Na]^+^, 100%, calculated for [C_16_H_20_^81^BrNO_2_+H]^+^ = 340.0, found 340.0 [M+H]^+^, 50%, calculated for [C_16_H_20_^79^BrNO_2_+Na]^+^ = 362.1, found 362.1 [M+H]^+^, 100%, HRMS (ESI^+^) calculated for [C_16_H_20_^79^BrNO_2_+H]^+^ = 338.0750, found [M+H]^+^ 338.0752, calculated for [C_16_H_20_^81^BrNO_2_+H]^+^ = 340.0730, found [M+H]^+^ 340.0731.

**7-Bromo-1-(4-(trifluoromethyl)benzyl)-1,5-dihydrobenzo[e][1,4]oxazepin-2(3H)-one (4b)**

Following general procedure 3, **4b** was obtained from was obtained from **3b** (1.50 g, 5.04 mmol) and chloroacetyl chloride (802 µL, 10.1 mmol). Purification by flash column chromatography (MeOH:CH_2_Cl_2_; 5:95) afforded the title compound as a yellow oil (1.76 g, 88%).

ν_max_ (film)/cm^-1^= 2910w (CH), 2860w (CH), 1690s (C=O), 1483m (C=C_arom_), 1417m (C=C_arom_); ^1^H NMR (400 MHz, CDCl_3_) δ 7.56-7.51 (3H, m), 7.47 (1H, d, ^4^*J*_HH_ = 2.3 Hz), 7.35 (2H, d, ^3^*J*_HH,_ Hz), 7.12 (1H, d, ^3^*J*_HH_ 8.6), 5.12 (2H, s), 4.47 (2H, s), 4.08 (2H, s); ^13^C NMR (126 MHz, CDCl_3_) δ 168.4, 141.3, 140.7, 133.6, 133.3, 131.3, 130.1 (q, ^2^*J*_CF_ = 33.1 Hz), 128.1, 125.8 (q, ^3^*J*_CF_ = 3.9 Hz), 121.9 (q, ^1^*J*_CF_ = 272.5 Hz), 122.7, 120.0, 67.5, 67.5, 50.4; LRMS (ESI^+^) calculated for [C_17_H_13_NO_2_^79^BrF_3_+H]^+^ = 400.0, found 400.0 [M+H]^+^, calculated for [C_17_H_13_NO_2_^81^BrF_3_+H]^+^ = 402.0, found 402.0 [M+H]^+^; HRMS (ESI^+^) calculated for [C_17_H_13_NO_2_^79^BrF_3_+H]^+^ = 400.0155, found 400.0155 [M+H]^+^, calculated for [C_17_H_13_NO_2_^81^BrF_3_+H]^+^ = 402.0134, found 402.0135 [M+H]^+^.

**7-Bromo-1-(4-methylbenzyl)-1,5-dihydrobenzo[e][1,4]oxazepin-2(3H)-one (4c; OX03146)**

Following general procedure 3, **4c** was obtained from **3c** (0.90 g, 2.95 mmol, 1.0 equiv.), chloroacetyl chloride (0.47 mL, 5.9 mmol, 2.0 equiv.) and NEt_3_ (0.82 mL, 5.9 mmol, 2.0 equiv.) for the acylation, followed by subsequent ester hydrolysis and concomitant cyclisation using aq. 10N NaOH (0.65 mL, 6.5 mmol, 2.2 equiv.). Purification by flash column chromatography (EtOAc:Petroleum; 1:9) afforded the compound as a pale yellow solid (0.53 g, 52%).

mp = 106-107 ^o^C; R_f_ = 0.40 (EtOAc:Petroleum; 1:9); ν_max_ (film)/cm^-1^= 2986w (CH), 2919w (CH), 2857w (CH), 1658s (C=O), 1485m (C=C_arom_), 1421m (C=C_arom_); ^1^H NMR (500 MHz, CDCl_3_) δ 7.50 (1H, dd, ^3^*J*_HH_ = 8.7 Hz, ^4^*J*_HH_ = 2.2 Hz), 7.43 (1H, d, ^3^*J*_HH_ = 2.2 Hz), 7.15 (1H, d, ^3^*J*_HH_ = 8.7 Hz), 7.11 (2H, d, ^3^*J*_HH_ = 8.1 Hz), 7.06 (2H, d, ^3^*J*_HH_ = 8.1 Hz), 5.04 (2H, s), 4.45 (2H, s), 4.05 (2H, s), 2.29 (3H, s); ^13^CNMR (500 MHz, CDCl_3_) δ 168.3, 141.6, 137.6, 133.8, 133.4, 133.1, 131.5, 129.5, 127.9, 123.2, 119.7, 67.7, 67.6, 50.5, 21.2; LRMS (ESI^+^) calculated for [C_17_H_15_^79^BrNO_2_+H]^+^ = 368.0, found 368.0 [M+H]^+^, 95%, calculated for [C_17_H_15_^81^BrNO_2_+H]^+^ = 370.0, found 370.0 [M+H]^+^_,_ 100%; HRMS (ESI^+^) calculated for [C_17_H_15_^79^BrNO_2_+H]^+^ = 368.0257, found 368.0257 [M+H]^+^, 95%, calculated for [C_17_H_15_^81^BrNO_2_+H]^+^ = 370.0236, found 370.0238 [M+H]^+^.

**General Procedure 4.**

To a solution of the desired bromide (1.0 equiv.) and the boronic acid (1.3 equiv.) in DMF (3 mL/mmol) in a microwave vial, was added aq. 1.5 M NaHCO_3_ (3.0 equiv.) and the vessel was degassed with argon for 10 minutes. Tetrakis(triphenylphosphine)palladium(0) (5 mol %) was then added, the microwave vial was sealed, and irradiated at 150°C for 15 min. The reaction mixture was allowed to cool, diluted with EtOAc (5 mL/mmol), washed with 0.5 M aq. LiCl (3 x 3mL/mmol). The combined organic layers were dried (Na_2_SO_4_), then concentrated *in vacuo*. The compound was then further purified by flash column chromatography (silica gel).

**1-(Cyclohexylmethyl)-7-(pyridin-3-yl)-1,5-dihydrobenzo[e][1,4]oxazepin-2(3H)-one (5a; OX02993)**

Following general procedure 4, **5a** was obtained from **4a** (250 mg, 0.74 mmol, 1.0 equiv.), 3‑pyridinylboronic acid (120 mg, 0.96 mmol, 1.3 equiv.), Pd(PPh_3_)_4_ (43 mg, 0.04 mmol, 5 mol%) and NaHCO_3_ (1.5mL, 2.6 mmol, 3.0 equiv.). Purification by flash column chromatography (EtOAc:Petroleum; 3:2) afforded the compound as an off-white solid (68 mg, 27%).

mp = 121-122 ^o^C; ; R_f_ = 0.25 (EtOAc:Petroleum; 3:2); ν_max_ (film)/cm^-1^ = 2926w (CH), 2909w (CH), 2857w (CH), 1672s (C=O), 1445m (C=C_arom_), 1432m (C=C_arom_); ^1^H NMR (500 MHz, CDCl_3_) δ 8.87 (1H, d, ^4^*J*_HH_ = 1.4 Hz), 8.63 (1H, d, ^4^*J*_HH_ = 4.7 Hz), 7.89 (1H, ddd, ^3^*J*_HH_ = 7.8 Hz, ^4^*J*_HH_ = 2.4 Hz, ^4^*J*_HH_ = 1.8 Hz), 7.67 (1H, dd, ^3^*J*_HH_ = 8.4 Hz, ^4^*J*_HH_ = 2.3 Hz), 7.57 (1H, ^4^*J*_HH_ = 2.3 Hz), 7.42-7.37 (2H, m), 4.75 (2H, s), 4.03 (2H, s), 3.86 (2H, d, ^3^*J*_HH_ = 6.8 Hz), 1.71-1.58 (6H, m), 1.19-1.1 (3H, m), 1.03-0.93 (2H, m); ^13^C NMR (126 MHz, CDCl_3_) δ 168.5, 149.1, 148.3, 142.9, 135.9, 135.3, 134.3, 130.2, 129.6, 128.8, 123.8, 121.8, 68.4, 68.0, 52.8, 36.8, 31.4, 26.4, 25.8; LRMS (ESI^+^) calculated for [C_21_H_24_N_2_O_2_+H]^+^ = 337.2, found 337.2 [M+H]^+^, 100%; HRMS calculated for [C_21_H_24_N_2_O_2_+H]^+ ­^= 337.1911, found 337.1909.

**7-(Pyridin-3-yl)-1-(4-(trifluoromethyl)benzyl)-1,5-dihydrobenzo[e][1,4]oxazepin-2(3H)-one (5b; OX03144)**

Following general procedure 4, **5b** was obtained from was obtained from **4b** (100 mg, 0.250 mmol) and 3-pyridyl boronic acid (40 mg, 0.326 mmol). Purification by flash column chromatography (MeOH:CH_2_Cl_2_; 7:93) afforded the title compound as a pale yellow solid (78 mg, 78%).

mp = 76-77 ^o^C; ν_max_ (film)/cm^-1^= 2985w (CH), 2976w (CH), 1668s (C=O), 1476m (C=C_arom_), 1435m (C=C_arom_); ^1^H NMR (400 MHz, CDCl_3_) δ 8.83 (1H, d, ^4^*J*_HH_ = 1.5 Hz), 8.62 (1H, dd, ^3^*J*_HH_ = 4.7 Hz, ^4^*J*_HH_ = 1.0 Hz), 7.88 (1H, ddd, ^3^*J*_HH_ = 8.0 Hz, ^4^*J*_HH_ = 2.5 Hz, ^4^*J*_HH_ = 1.6 Hz) 7.65 (1H, dd, ^3^*J*_HH_ = 8.3 Hz, ^4^*J*_HH_ = 2.5 Hz) 7.58-7.55 (3H, m), 7.41 (2H, m), 7.35 (2H, d, ^3^*J*_HH_ = 8.3 Hz), 5.20 (2H, s), 4.61 (2H, s), 4.14 (2H, s); ^13^C NMR (100 MHz, CDCl_3_) δ 168.6, 149.2, 148.2, 142.2, 141.0, 136.6, 135.1, 134.3, 130.3, 130.2 (q, ^3^*J*_CF_ = 33.8 Hz), 129.5, 128.9, 128.9, 128.1, 125.9 (q, ^3^*J*_CF_ = 3.7 Hz), 123.1 (q, ^2^*J*_CF_ = 271.4 Hz), 121.8, 68.2, 67.7, 50.6; LRMS (ESI^+^) calculated for [C_22_H_17_N_2_O_2_F_3_+H]^+^ = 399.1, found 399.1 [M+H]^+^, 100%; HRMS (ESI^+^) calculated for [C_22_H_17_N_2_O_2_F_3_+H]^+^ = 399.1315, found 399.1312 [M+H]^+^.

**1-(4-Methylbenzyl)-7-(pyridin-4-yl)-1,5-dihydrobenzo[e][1,4]oxazepin-2(3H)-one (5c; OX03153)**

Following general procedure 4, **5c** was obtained from was obtained from **4c** (1.00 g, 2.90 mmol) and 3-pyridyl boronic acid (463 mg, 3.77 mmol). Purification by flash column chromatography (MeOH:CH_2_Cl_2_; 6:94) afforded the title compound as a yellow oil (921 mg, 92%).

mp = 117-118 ^o^C; ν_max_ (film)/cm^-1^= 2922w (CH), 2860w (CH), 1664s (C=O), 1475m (C=C_arom_), 1434m (C=C_arom_); ^1^H NMR (400 MHz, CDCl_3_) δ 8.81 (1H, d, ^4^*J*_HH_ = 1.6 Hz), 8.59 (1H, dd, ^3^*J*_HH_ = 4.8 Hz, ^4^*J*_HH_ = 1.2 Hz), 7.84 (1H, ddd, ^3^*J*_HH_ = 8.0 Hz, ^4^*J*_HH_ = 2.4 Hz, ^4^*J*_HH_ = 1.5 Hz), 7.60 (1H, dd, ^3^*J*_HH_ 8.4 Hz, ^4^*J*_HH_ = 2.2 Hz), 7.51 (1 H, d, ^4^*J*_HH_ = 2.2 Hz), 7.41-7.34 (3H, m), 7.16 (2H, d, ^3^*J*_HH_ = 8.1 Hz), 7.08 (2H, d, ^3^*J*_HH_ = 8.1 Hz), 5.11 (2H, s), 4.58 (2H, s), 4.10 (2H, s), 2.28 (3H, s); ^13^C NMR (100 MHz, CDCl_3_) δ 168.4, 149.0, 148.1, 142.5, 137.4, 136.2, 135.2, 134.2, 134.0, 130.2, 129.5, 129.2, 128.6, 127.8, 123.8, 122.1, 68.1, 67.8, 50.5, 21.2; LRMS (ESI^+^) calculated for [C_22_H_20_N_2_O_2_+H]^+^ = 345.2, found 345.2, [M+H]^+^, 100%, calculated for [C_22_H_20_N_2_O_2_+Na]^+^ = 367.1, found 367.2 [M+Na]^+^, 20%; HRMS (ESI^+^) calculated for [C_22_H_20_N_2_O_2_+H]^+^ 345.1598, found 345.1598 [M+H]^+^.

**1-(4-Methylbenzyl)-7-(pyridin-4-yl)-1,5-dihydrobenzo[e][1,4]oxazepin-2(3H)-one (5d; OX02983)**

Following general procedure 4, **5d** was obtained from **4c** (300 mg, 0.87 mmol, 1.0 equiv.), 4‑pyridinylboronic acid (160 mg, 1.13 mmol, 1.3 equiv.), Pd(PPh_3_)_4_ (46 mg, 0.04 mmol, 5 mol%) and 1.5 M NaHCO_3_ (1.3 mL, 2.6 mmol, 3.0 equiv.). Purification by flash column chromatography (EtOAc:Petroleum; 3:2) afforded the compound as an off-white solid (130 mg, 43%).

mp = 154-155 ^o^C; R_f_ = 0.65 (EtOAc:Petroleum; 3:2); ν_max_ (film)/cm^-1^ = 2974w (CH), 2925w (CH), 2862w (CH), 1666s (C=O), 1431m (C=C_arom_), 1413 (C=C_arom_); ^1^H NMR (500 MHz, CDCl_3_) δ 8.67 (1H, ^4^*J*_HH_ = 4.9 Hz), 7.67 (1H, dd, ^3^*J*_HH_ = 8.4 Hz, ^4^*J*_HH_ = 2.2 Hz), 7.57 (1H, d, ^4^*J*_HH_ = 2.2 Hz), 7.49-7.46 (2H, m), 7.40 (1H, d, ^3^*J*_HH_ = 8.4 Hz), 7.16 (2H, d, ^3^*J*_HH_ = 8.0 Hz), 7.08 (2H, d, ^3^*J*_HH_ = 8.0 Hz), 5.12 (2H, s), 4.60 (2H, s), 4.11 (2H, s), 2.29 (3H, s); ^13^C NMR (126 MHz, CDCl_3_) δ 168.5, 150.6, 146.8, 143.4, 137.6, 136.4, 134.0, 130.3, 129.5, 129.2, 128.6, 127.9, 122.1, 121.5, 76.9, 68.2, 67.9, 50.5, 21.3; LRMS (ESI^+^) calculated for [C_22_H_20_N_2_O_2_+H]^+^ = 345.2, found 345.2 ([M+H]^+^ 100%), calculated for [C_22_H_20_N_2_O_2_+Na]^+^ = 367.1, found 367.2 [M+Na] 10%; HRMS (ESI^+^)

calculated for [C_22_H_20_N_2_O_2_+H]^+^ = 345.1598, found [M+H]^+^ 345.1597.

**2a ^1^H NMR Spectrum**

**2a ^13^C NMR Spectrum**

**2b ^1^H NMR Spectrum**

**2b ^13^C NMR Spectrum**

**2c ^1^H NMR Spectrum**

**2c ^13^C NMR Spectrum**

**3a ^1^H NMR Spectrum**

**3a ^13^C NMR Spectrum**

**3b ^1^H NMR Spectrum**

**3b ^13^C NMR Spectrum**

**3c ^1^H NMR Spectrum**

**3c ^13^C NMR Spectrum**

**4a ^1^H NMR Spectrum**

**4a ^13^C NMR Spectrum**

**4b ^1^H NMR Spectrum**

**4b ^13^C NMR Spectrum**

**4c ^1^H NMR Spectrum**

**4c ^1^H NMR Spectrum**

**5a ^1^H NMR Spectrum**

**5a ^13^C NMR Spectrum**

**5b ^1^H NMR Spectrum**

**5b ^13^C NMR Spectrum**

**5c ^1^H NMR Spectrum**

**5c ^13^C NMR Spectrum**

**5d ^1^H NMR Spectrum**

**5d ^1^C NMR Spectrum**
